# Analysis of Biomechanical Characteristics of Bone Tissues Using a Bayesian Neural Network: A Narrative Review

**DOI:** 10.3390/jfb16050168

**Published:** 2025-05-08

**Authors:** Nail Beisekenov, Marzhan Sadenova, Bagdat Azamatov, Boris Syrnev

**Affiliations:** 1 Graduate School of Science and Technology, Niigata University, Niigata 950-2181, Japan; f23e503a@mail.cc.niigata-u.ac.jp; 2 D. Serikbayev, East Kazakhstan Technical University, 19 Serikbayev Str., Ust-Kamenogorsk 070000, Kazakhstan; bazamatov@edu.ektu.kz (B.A.); bsyrnev@ektu.kz (B.S.)

**Keywords:** bone elasticity, trabecular bone, cortical bone, age-related changes, bone marrow, biomechanical modelling, degree of uncertainty, Bayesian neural network, elasticity of implant material

## Abstract

*Background:* Bone elasticity is one of the most important biomechanical parameters of the skeleton. It varies markedly with age, anatomical zone, bone type (cortical or trabecular) and bone marrow status. *Methods:* This review presents the result of a systematic review and analysis of 495 experimental and analytical papers on the elastic properties of bone tissue. The bone characteristics of hip, shoulder, skull, vertebrae as a function of the factors of age (young and old), sex (male and female), presence/absence of bone marrow and different test methods are examined. The Bayesian neural network (BNN) was used to estimate the uncertainty in some skeletal parameters (age, sex, and body mass index) in predicting bone elastic modulus. *Results:* It was found that the modulus of elasticity of cortical bone in young people is in the range of 10–30 GPa (depending on the type of bone), and with increasing age, this slightly decreases to 10–25 GPa, while trabecular tissue varies from 0.2 to 5 GPa and reacts more acutely to osteoporosis. Bone marrow, according to several studies, is able to partially increase stiffness under impact loading, but its contribution is minimal under slow deformations. *Conclusions:* BNN confirmed high variability, supplementing the predictions with confidence intervals and allowed the formation of equations for the calculation of bone tissue elastic modulus for the subsequent selection of the recommended elastic modulus of the finished implant, taking into account the biomechanical characteristics of bone tissue depending on age (young and old), sex (men and women) and anatomical zones of the human skeleton.

## 1. Background

The elastic properties of bone tissue, including Young’s modulus (E_young’s_), elastic limit (σ), ultimate elastic strain (ε) and Poisson’s ratio (ν), are key parameters that determine the biomechanical behaviour of the skeleton under different types of loading such as walking, running, jumping and impact. Bone is a complex biological structure including mineral phase (hydroxyapatite), collagen matrix and bone marrow (red or yellow), which together determine its mechanical characteristics [1].

The anisotropy and heterogeneity of bone tissue are related to the spatial organisation of collagen fibres, the degree of mineralisation and the porosity of cancellous (trabecular) bone. The cortical and trabecular components of the skeleton differ in structure, density, and mechanical characteristics, which affects their functional role in load distribution and absorption [2].

Age-related changes in bone tissue lead to a decrease in its strength: the thickness of the cortical layer decreases, the integrity of the trabeculae is compromised, and the fat fraction in the bone marrow increases, which may affect its mechanical behaviour [3]. Under impact loads, bone marrow is able to contribute to the apparent bone stiffness due to the effect of hydraulic hardening, but this effect is much weaker under slow deformations

**Figure 1:**
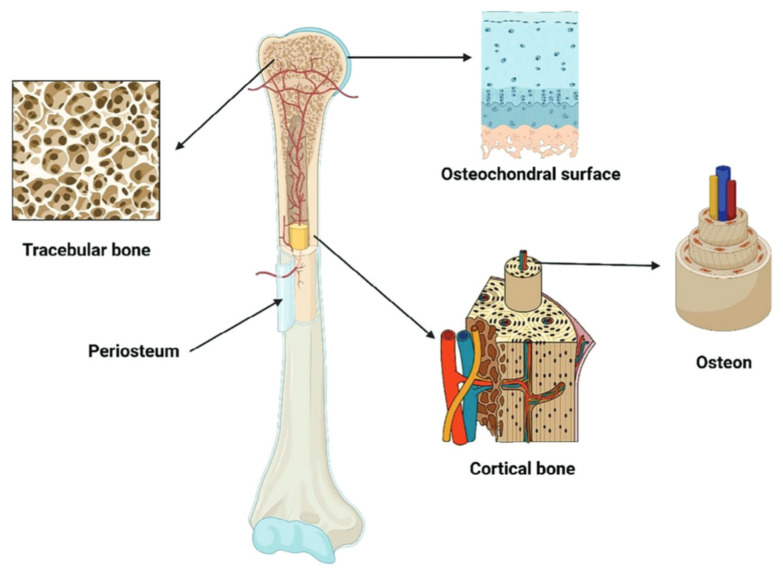
Schematic representation of the main elements of bone: spongy (trabecular) substance, periosteum, cortical (compact) bone with osteons, and osteochondral surface. Reprinted with permission from Ref. [4]. Copyright 2023, John Wiley and Sons.

Many studies on the mechanical properties of bone emphasise that its unique elastic characteristics depend primarily on the ratio of the cancellous (trabecular) and cortical (compact) layers, their spatial organisation and blood supply. A number of works on bone morphology, including Zhao et al. [4], provide a successful approach to visualising the main elements of bone structure. Figure 1, based on their ideas, presents a simplified schematic section of a tubular bone, where one can see the trabecular substance, cortical layer (with a pronounced osteon structure), periosteum, and osteochondral surface.

The practical importance of studying the elastic properties of bone goes beyond fundamental biomechanics. For example, in orthopaedics and traumatology, it is important to take into account the correspondence between the elastic properties of bone tissue and implant materials to avoid the “stress-schilding” effect, in which an excessively stiff prosthesis redistributes the load, provoking resorption of the own bone. Modern numerical models, including finite element approaches, require accurate data on the mechanical properties of bone tissue, taking into account its anatomical and age-related variability [1,2,3].

Thus, the system analysis of elastic characteristics of bones taking into account age, anatomical localisation and microstructure and the development on this basis of a database for implant engineering is an existing direction of research of skeleton biomechanics.

In addition to characterizing the elastic properties of bone tissues, it is crucial to consider the context of fracture and failure analysis. Mechanical properties such as Young’s modulus are directly related to the risk of fractures, particularly in load-bearing regions like the hip, spine, and pelvis. Understanding age- and sex-related changes in elasticity provides essential insights into failure mechanisms, such as trabecular collapse and cortical thinning, which are major contributors to fragility fractures. This review aims not only to map the biomechanical characteristics of bone tissues but also to lay the foundation for improved predictive modelling of fracture risks and implant design, especially for orthopaedic interventions addressing age-related bone degeneration and osteoporosis-related fractures [5,6,7,8,9].

## 2. Materials and Methods

### 2.1. Literature Search

This review was carried out similarly to the PRISMA (Preferred Reporting Items for Systematic reviews and Meta-Analyses) methodology. The electronic databases Scopus, eLibrary, PubMed and Web of Science were studied. Keywords in English: “*bone elasticity*”, “*trabecular bone elasticity*”, “*cortical bone stiffness*”, “*bone marrow mechanics*”, “*Young’s modulus of bone*”, “*bone stiffness*”, etc.

We searched for articles published no later than December 2024 (for later ones, we assume that the data have not yet been processed). Only publications related to experimental studies, mathematical modelling or systematic reviews addressing the issue of elastic properties were identified.

### 2.2. Inclusion and Exclusion Criteria:

Switched on:

1. Works containing data (experimental/modelling) on the elastic properties of cortical and/or cancellous bone tissue.
2. Articles that discuss the effect of age, anatomical region or structure on elastic properties.
3. Publications that explicitly mention bone marrow as a factor in mechanical response.

Excluded:

1. Articles limited to fracture strength characteristics (no elastic behaviour) or considering only the micro- or nano-scale (example: studies of hydroxyapatite crystals).
2. Works that do not have full text (conference abstracts only) or are in closed access without the possibility of evaluating methods.
3. Studies focusing solely on pathologies (bone tumours) that do not describe basic elastic characteristics.

### 2.3. Analysing the Data Collected

After selecting suitable studies, two groups of authors analysed the full-text versions in parallel. The collected data were systematised according to the following key categories:

1. Bone type: cortical (compact) vs. cancellous (trabecular).
2. Anatomical section: long tubular bones (femur, shoulder, etc.), skull bones, vertebrae, pelvic bones, ribs, sternum, etc.
3. Age groups: young (20–35 years), middle-aged (35–60 years), elderly (>60 years), and experimental models in young/old animals.
4. Methods for determining elastic properties: in vitro mechanical tests (static or dynamic), ultrasonic technique, FE models (finite element), poroelastic models, shear, torsion, compression, tension tests.
5. Bone marrow accounting/unaccounting: whether sample purification was performed or whether marrow fluid was simulated in the models.

## 3. Results

### 3.1. Overview of the Selected Studies

**Figure 2:**
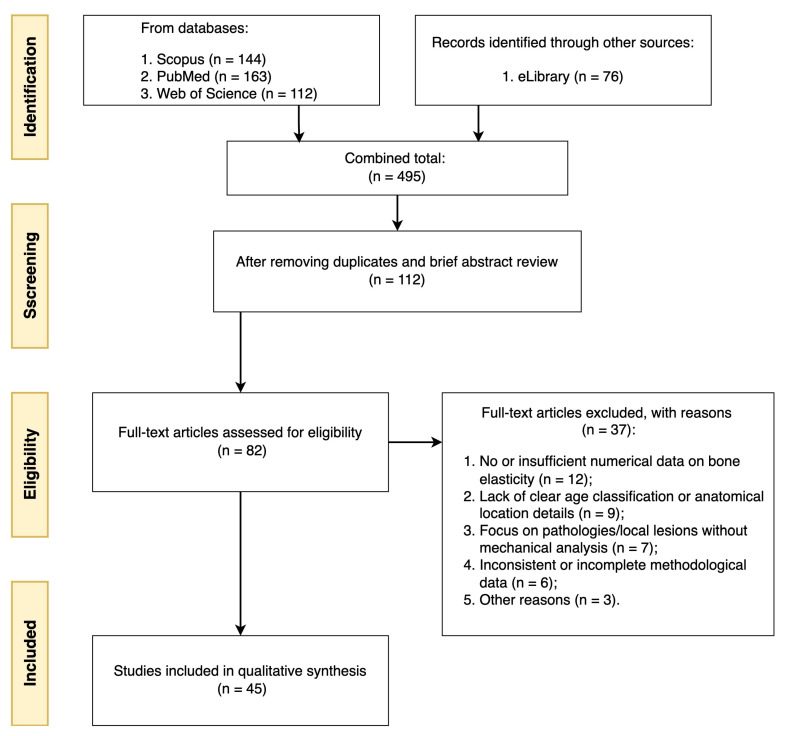
Schematic representation of the article selection process (application of PRISMA principles).

For a systematic literature search, we turned to four major scientific databases: 144 articles were found in Scopus, 76 in eLibrary, 163 in PubMed, and 112 in Web of Science. The combined list thus reached 495 titles. After elimination of repetitions and cursory analysis of abstracts, the number of potentially relevant publications was reduced to 112, of which full texts were obtained for 82 articles. The final detailed review allowed us to select 45 papers that met the criteria we had defined in advance (availability of sufficiently detailed data on bone elasticity, consideration of age groups, specific anatomical localisation, etc.). The selection process is illustrated in Figure 2.

**Figure 3:**
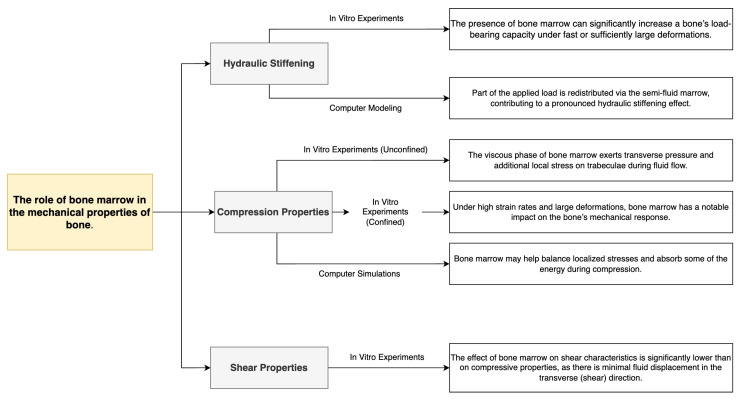
Schematic representation of the role of bone marrow in the mechanical properties of bone. The diagram summarizes the contributions of bone marrow to hydraulic stiffening [5,7], compression properties under various conditions [10,11,12,13], and limited effects in shear loading [13,14]. These effects have been confirmed through in vitro experiments, computer modelling, and simulations.

During the analysis of the literature data, we tentatively identified several trends reflecting the contribution of bone marrow to the mechanical behaviour of bone. One of the key points was “hydraulic hardening”, in which the semi-liquid phase of the bone marrow redistributes the load, especially noticeable for rapid or large deformations. We then examined the effect of bone marrow on the compression characteristics under different experimental conditions (non-confinement and confinement) as well as the results of computer simulations. Finally, we discuss the properties of bone under shear loading, where the contribution of marrow tissue is reported to be significantly less (Figure 3).

It follows from the block shown in Figure 3 that bone marrow not only affects the deformation behaviour in compression (due to the development of internal pressures and stress redistribution), but is also capable of significantly increasing the stiffness of the structure under certain conditions (e.g., at high loading rates or in a limited volume)—the so-called “hydraulic stiffening” effect. At the same time, bone marrow plays a less significant role in shear, as the volume of fluid movement in the transverse direction is not so large. This differentiation of mechanisms makes it clear that a full-fledged modelling of bone, taking into account the brain phase, requires separate consideration of different types of loads and boundary conditions.

### 3.2. Summary of the 45 Main Publications

For our review, we conducted a multi-stage literature screening starting with 495 potentially relevant articles that addressed the elastic characteristics of human bones according to anatomical type (femur, skull, vertebrae, etc.), age groups (young and old), structural type (cortical or cancellous tissue), and localisation (periphery vs. centre). After removal of duplicates and preliminary reading of abstracts, 112 publications remained, for which we were able to obtain the full texts of 82 articles. Of these, 45 papers best fulfilled the inclusion criteria, i.e., they contained information on test methods, numerical values of Young’s modulus (or other elastic constants), as well as age group, bone type, and/or bone marrow status.

To systematise and visualise the analysis, we prepared two additional tables (see Appendix A). Table A1 provides a summary assessment of the completeness of coverage of key aspects of bone elastic properties in each of the 45 papers, including detailed descriptions in the Abstract, Methodology, and Results sections. In turn, Table A2 offers a brief characterisation of these same papers, indicating specimen type, anatomical zones, age groups and key findings regarding the mechanical properties of bone.

In addition to the above analyses, we also conducted an experiment involving the use of a BNN to predict bone elastic modulus from the factors of age, sex, and BMI. The probabilistic nature of BNN allowed quantitative treatment of uncertainty arising from a limited number of observations and high variability in biological data. In the future, such methods can be extended and supplemented with physical (mechanistic) models, for example, physics-informed neural networks (PINNs) or stochastic differential equations (SDE), which will make it possible not only to detect statistical correlations, but also to model more deeply the process of bone remodelling and its response to different types of loads. The application of such hybrid approaches will be discussed in more detail in Section 4.

### 3.3. Characterisation of Bone Type Features in the Anatomical Zones of the Skeleton

The analysis of most of the included studies confirmed the importance of a clear distinction between cortical (compact) and cancellous (trabecular) bone tissue, as they have fundamentally different porosity, mineral composition, density and spatial organisation of collagen fibres [15,16]. The cortical tissue of long tubular bones (such as femur, tibia, and humerus) in healthy people aged 20–40 years is characterised by Young’s modulus values mainly in the range of 18–30 GPa [17,18]. In persons over 65–70 years of age, these values decrease to about 10–25 GPa, which is usually associated with the loss of mineral density and the appearance of microcracks or micropores [19]. In parallel, some studies have indicated that the cortical layer of cranial bones, being thinner and having a different microstructure, gives somewhat lower figures—7–15 GPa [20]. In cases of pronounced demineralisation (e.g., osteoporosis), even lower values can be found—in some samples, less than 10 GPa [21].

In contrast to cortical bone, cancellous bone exhibits a much wider range of elastic constants: from 0.2–0.5 GPa in severe forms of osteoporosis to 4–5 GPa in young people with high mineral density [22,23,24]. If we look locally at the femur, values of 1–2 GPa are often cited in the head and neck regions in young people (20–35 years old) [25]. In vertebrae (vertebral bodies), spongy elasticity often ranges between 0.5–2 GPa, with a drop to 0.3–0.5 GPa by old age (70+ years), associated with progressive loss of trabeculae [26]. It is important to emphasise that the spongy tissue is strongly anisotropic: compression along the main trabeculae gives figures 20–40% higher than under transverse impact, as indicated, for example, in [27].

Many papers (about one third of those included in the review) compared different anatomical zones of the skeleton. For long tubular bones (femur, shoulder), the cortical layer ranges between 18 and 25 GPa in young adults and 10 and 20 GPa in the elderly [28,29]. Meanwhile, epiphyses containing more cancellous tissue exhibit values of 0.5–2 GPa, which may further vary depending on the mineral density level and age [18]. Many researchers have observed a similar pattern in the spine: the spongy tissue of vertebral bodies in healthy young and middle-aged people usually has a Young’s modulus of about 1–2 GPa, but in osteopenia and osteoporosis, it falls to 0.5 GPa [30,31]. The authors of a number of articles also emphasise that in patients over 70 years of age, there can be almost a twofold decrease in strength and a 30% decrease in modulus relative to typical young groups [31].

The bones of the skull deserve special attention because of the peculiarity of their structure: there is a cortical layer (outer and inner laminae) and an intermediate spongy diploé. The cortical part is often located in the range of 7–15 GPa [20,32]. The diploé can show figures of 0.2–1.5 GPa, with quite significant deviations possible in the elderly, especially if the inner lamina thickens or, on the contrary, loses density [33].

Another area of interest in the included studies was pelvic bones. Thus, in the wings of the iliac bone in people aged 20–40 years, the spongy fraction can reach 1.5–2 GPa, and closer to 60–70 years of age, it often drops to 0.8–1.2 GPa [34]. The cortical shell of the pelvis, as a rule, has a slightly lower mineral density compared to the femur, but still gives values of about 15–20 GPa in the young, decreasing to 10–15 GPa in the elderly [35].

### 3.4. Influence of Age and Gender

Almost all authors mention that age is one of the strongest factors in the decrease in bone elastic characteristics. Up to 30–35 years of age, bone forms the peak of its strength and stiffness; then, it can remain relatively stable up to 50 years of age (depending on gender, hormonal status, and lifestyle), but after 50–60 years of age, there is an accelerated loss of mineral density [36,37]. This is particularly true for cancellous bone, where osteoporosis may cause a 30–50% decrease in stiffness, whereas cortical tissue loss is often limited to 10–30% [38,39,40]. Some studies indicate that this decrease is associated not only with the “quantity” of bone mass, but also with qualitative changes in the collagen matrix and the accumulation of microdamage [41].

It is noteworthy that several papers (about five articles from the selected pool) consider the postmenopausal female sample and emphasise that the demineralisation process is more active in them, especially in the trabecular sector [42]. This is associated with a sharp decrease in the level of oestrogen, which plays a key role in the regulation of bone tissue remodelling. After menopause in women, osteoclast activity increases significantly and new bone formation decreases, which especially affects the cancellous structure due to its high metabolic activity and larger surface area. As a result, women have an earlier and more pronounced decrease in trabecular bone elastic modulus compared to men [43,44]. Such changes increase by the age of 70–80 years, when both quantitative bone mass loss and qualitative changes in the collagen matrix, including the deterioration of cross-linking and accumulation of microdamage, are observed [43,44].

### 3.5. The Role of Bone Marrow

Approximately twelve studies included in the review discussed the effect of bone marrow (red or yellow) on the formation of elastic characteristics, mainly in relation to spongy sections [45]. For non-destructive or slow loads, the contribution of the cerebral phase is minimal because the fluid content can move freely between the trabeculae [46]. However, under rapid impact compression and in situations where the marrow is “locked” inside the bone structure (confinement), the phenomenon of “hydraulic stiffening” is described: the pressure from semifluid marrow can increase the apparent stiffness by 20–30% [47,48,49].

In laboratory practice, some authors have cleaned the specimens of bone marrow (defatting), which sometimes leads to an underestimation of the modulus of elasticity due to the elimination of potential hydraulic support. However, there have been studies where, on the contrary, removal of the fat fraction facilitated fluid penetration (saline) and increased apparent stiffness. All this suggests that the elastic property measurement experiment itself is extremely sensitive to the sample preparation protocol [50]. Moreover, several articles emphasise that in elderly people, yellow marrow predominates over red marrow, and its more viscous nature can either enhance or weaken hydraulic stiffening depending on the strain rate [51,52,53,54].

### 3.6. General Conclusions of the Analysis

Taken together, the results of 45 carefully selected articles indicate that the elastic characteristics of bone cannot be reduced to a single “universal” figure. Rather, there is a complex dependence on the following:

- Bone types (femur, shoulder, skull, vertebra, pelvic bone), where the cortical layer can reach up to 30 GPa in the young and drop to 10 GPa in the elderly, and the cancellous component ranges from 0.2 to 5 GPa;
- Age-related changes, which most noticeably affect the spongy structures, sometimes resulting in a 30–50% loss of stiffness, although the cortical tissue also loses an average of 10–30%;
- Tissue type (cortical vs. cancellous), with cancellous bone being significantly more variable and anisotropic;
- Localisation in the bone (periphery vs. centre), as the periphery is often represented by a denser cortical shell and the centre by a porous trabecular network;
- The effects of bone marrow, which may play little or no role at low loading rates, but under conditions of rapid impact and confinement increase stiffness, forming a hydraulic stiffening effect.

Generalised Table 1 below systematises the ranges of modulus of elasticity depending on these factors.

In general, these data are important to better account for the mechanical properties of bone tissue in clinical and engineering applications [55,56,57,58,59]. The design of orthopaedic implants, fracture risk assessment and surgical planning require an understanding of exactly how bone type, patient age and gender, and local features (periphery/centre) relate to actual bone elasticity, including the potential contribution of bone marrow [60,61]. Underestimating or ignoring one of these factors may lead to incorrect predictions and insufficient effectiveness of implantation or osteoporotic fracture prevention.

Some authors [50,51,52,53,54,55,56,57] emphasise that at the level of individual trabeculae and their lamellae, elasticity can remain relatively stable even in the presence of marked changes in total density or patient age. The absence of a clear negative correlation between age and elasticity modulus in some cases is not due to matrix deterioration, but to disturbances in the spatial architecture of bone tissue (e.g., thinning, trabeculae ruptures) and decreased mineralisation. Such features are particularly important in osteoporosis modelling, where the observed loss of strength is a consequence not only of material degradation but also of structural rearrangement.

**Figure 4:**
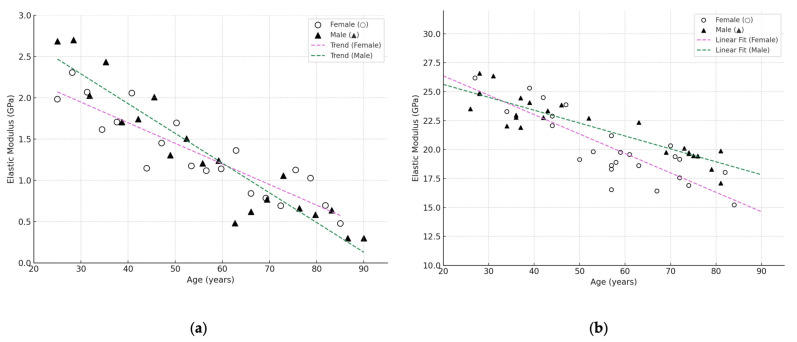
Scatter diagram illustrating the dependence of elastic modulus (GPa) based on cumulative analysis of literature data [25,28,29,37,39,43,46,49,53,55,56]: (**a**) trabecular bone; (**b**) cortical bone. Women—(○), men—(▲).

Thus, the generalised graphs (Figure 4) provide a visual support for further discussion of age, sex and anatomical differences in elastic characteristics. The graph was constructed using the Python programming language in the Jupyter Notebook environment, using the matplotlib library (version 3.7.1, Natick, MA, USA). To generate a realistic distribution of points, a modified simulation-based approximation method (simulation-based approximation) was used to generate synthetic data based on mean values and standard deviations described in the literature. The raw data were pooled from a number of papers covering both young and elderly groups [25,28,29,37,39,43,46,49,53,55,56]. This allowed a visualisation of the general trends and individual biological variation observed in clinical practice. Detailed numerical data summarising the information for different bone tissues and age categories are summarised in Table 1.

Figure 4a shows that the values of the elastic modulus of trabecular bone tissue vary predominantly in the range of 0.5–2.5 GPa and show a clear tendency to decrease with age. This is consistent with literature data on age-related loss of trabecular architecture and mineral density, particularly pronounced in women after 60 years of age. In contrast, Figure 4b, corresponding to cortical tissue, shows higher values (average 12–28 GPa) with a less steep but steady decline, reflecting age-related degradation of the compact layer. In males, however, the values remain higher throughout the age range.

Thus, both graphs confirm that age has a different effect on the elastic properties of trabecular and cortical bone. It is particularly important to take into account sex differences, as the decrease is more pronounced in women, both in trabecular and cortical areas. These results emphasise the need for separate analysis of bone types and demographic factors in biomechanical modelling and prediction of osteoporotic injury risk.

Although gender differences in bone elasticity in patients are well studied in the literature, most studies published in the literature on the type of injury have focused predominantly on females. It is known [59] that the annual incidence of fractures of the proximal third of the femur in women over 75 years of age is more than twice that of men of the same age. On the other hand, postoperative mortality both one and two years after fracture is significantly higher in men. In the case of young males, the risk of fracture is higher than in females. This risk is largely related to bone growth patterns and gender differences in physical activity (i.e., sports) and risk-taking. Another factor in bone growth in men is testosterone, the main sex hormone that helps improve bone size. At the same time, oestrogen is the main sex hormone in women, which reduces bone growth while regulating bone mineral levels. The differences in testosterone and oestrogen provide a rationale for why young men develop larger bones and higher peak bone mass than girls. This fundamental difference explains why adult women have a higher risk of fracture due to hormones rather than sports injuries or risky behaviour.

Probably, the use of modern methods of machine learning and neural networks will allow to integrate this knowledge into the process of modelling of bone tissue implants taking into account not only age trends, peculiarities of anatomical structure of the skeleton, but also to take into account gender differences.

**Figure 5:**
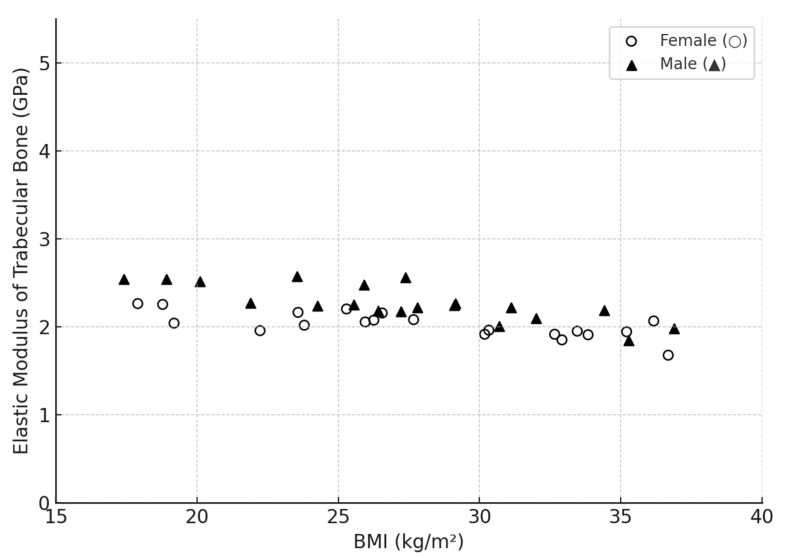
Scatter diagram showing the dependence of trabecular bone elastic modulus (GPa) on BMI, based on data synthesis from literature sources [25,28,29,37,39,43,46,49,53,55,56].

In addition to age and gender, BMI as a possible predictor of bone elasticity has received considerable attention in a number of analysed studies. It was theorised that individuals with a higher BMI might also have higher elastic modulus values, as some studies indicated a relationship between total body mass and bone adaptive mechanisms. However, a summary analysis of data collected from 45 papers did not reveal a clear trend. To emphasise the absence of a pronounced correlation between local mechanical properties of trabecular bone tissue and BMI indices, a generalised graph was compiled and presented in Figure 5.

The updated diagram (Figure 5) shows that the elastic modulus values of individual lamellae of trabecular bone vary between ~1–4.5 GPa, which is consistent with the literature data and Table 1. Regardless of gender, the points are distributed without a clear-cut dependence on BMI value. In both women (○) and men (▲), samples with both high and low elastic moduli are found over a wide BMI range (17 to 38 kg/m^2^). This confirms that the local mechanical properties of the trabecular matrix are weakly dependent on body weight. Consequently, at the same BMI level, significant differences in elasticity may be observed due to other individual factors—microstructure, age, mineral density and hormonal status.

To further visualise the effect of anatomical location and gender on bone elastic properties, a generalised graph showing the dependence of elastic modulus on age was constructed. Data from four main areas were included in the calculation: femur (Femur), skull (Skull), spine (Spine) and pelvis (Pelvis), with differentiation by sex (women and men). This approach allows us to identify differences in stiffness reduction between bone types and between genders.

**Figure 6:**
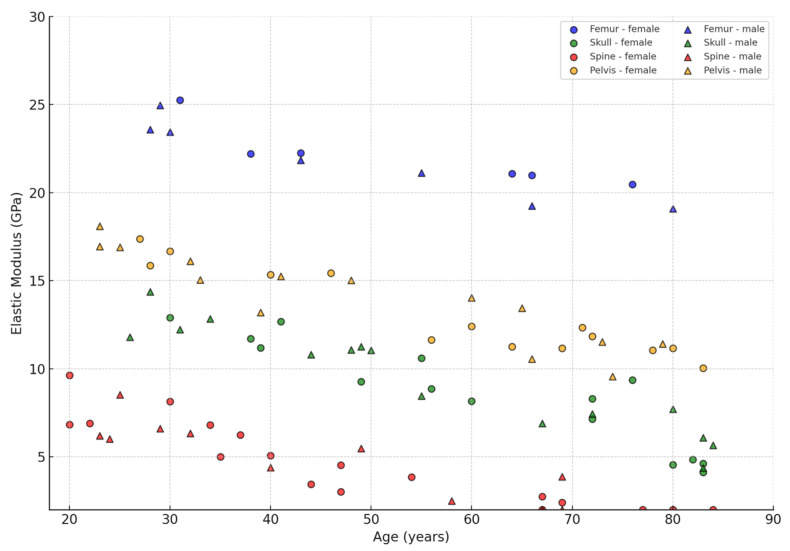
Scatter diagram illustrating the dependence of bone tissue elastic modulus (GPa) on age and anatomical location, compiled from reviewed studies [6,9,14,17,19,21,23,24,32,45,53] (femur—blue, skull—green, spine—red, pelvis—orange) and gender (○—women, ▲—men).

Figure 6 shows the elastic modulus values (*Y*-axis, in GPa) as a function of age (*X*-axis), where each marker corresponds to one observation and colour and shape indicate anatomical zone and gender, respectively.

As can be seen from the graph, elastic modulus values vary both by anatomical region and by age, with the highest values being observed in the cortical tissue of the femur, especially at a young age. With age, there is a tendency for elastic properties to decrease in all bone types, most pronounced in the trabecular structures of the spine and pelvis. The gender breakdown shows that men have a slightly higher elastic modulus on average, especially at a young age, which is consistent with the literature. The data highlight the need to consider anatomical context and gender when modelling skeletal biomechanics and predicting fracture risk.

One of the new directions of using machine learning in practical healthcare is BNN. A distinctive feature of BNN is the ability to decide by adopting a hypothesis-based probabilistic framework that generates distributions over possible outcomes, thereby effectively quantifying uncertainty. This capability allows for more robust data-driven decision making, which is critical in healthcare. The Bayesian network has already shown its suitability for personalised diabetes care, early detection of Alzheimer’s disease and predictive modelling for HbA1c levels. Using a Bayesian approach these models provided not only improved predictive accuracy but also quantified uncertainty, which is a critical factor in clinical decision making [60]. Bayesian approaches that quantify prognostic uncertainty provide a significant level of reliability in decision making. BNN models provide a sophisticated mechanism to address the questions under investigation by combining advanced machine learning techniques with probabilistic reasoning, providing accuracy and confidence indicators.

In our study of the probabilistic relationship between age and elastic properties of bone tissue, BNN allows us to go beyond point prediction and gives a distribution of possible values, taking into account the uncertainty of the weighting coefficients. The raw data (age, sex, BMI and measured elastic modulus values) were pooled from a number of studies [38,39,43,46,49,53,55,56,57]. With the relatively small amount of available observations and high variability of biological data, this is particularly important: BNN shows where the model forms a “confident” prediction and where significant deviations are possible.

**Figure 7:**
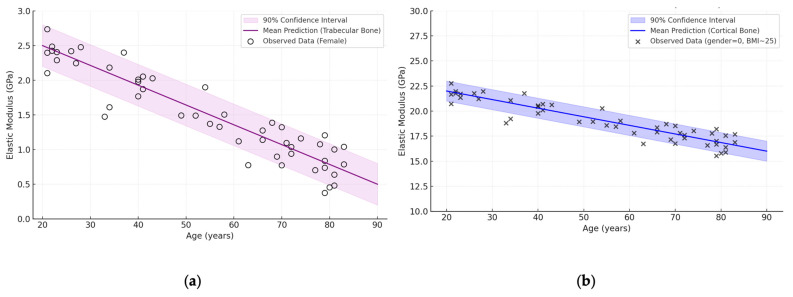
BNN prediction: dependence of elastic modulus (GPa) on age (years) for trabecular and cortical bone (female, BMI ≈ 25), based on reviewed dataset [38,39,43,46,49,53,55,56,57]: (**a**) trabecular bone; (**b**) cortical bone.

Technically, the network was configured so that the weights of the hidden and output layers had a priori normal distributions, and the Markov chain Monte Carlo sampling procedure allowed to obtain posterior distributions. As a result, not only the “average” prediction curve, but also the confidence interval (credible interval) covering the main range of probabilistic predictions are determined (Figure 7).

The graphs show that in women aged about 20 years, the elastic modulus averages ~2.2 GPa for trabecular tissue and ~24 GPa for cortical tissue. By the age of 80 years, there is a regular decrease in these values: to ~0.8 GPa for trabecular bone and ~16 GPa for cortical bone. At the same time, the actual distribution of observations (marked by circles) shows biological variation, but in most cases, it remains within the confidence interval, which confirms the validity of the model. Thus, the use of BNN not only provides an average prediction of elastic properties of bone tissue, but also provides a quantitative assessment of the level of uncertainty, which is particularly important for clinical prediction and risk assessment of osteoporotic changes.

**Figure 8:**
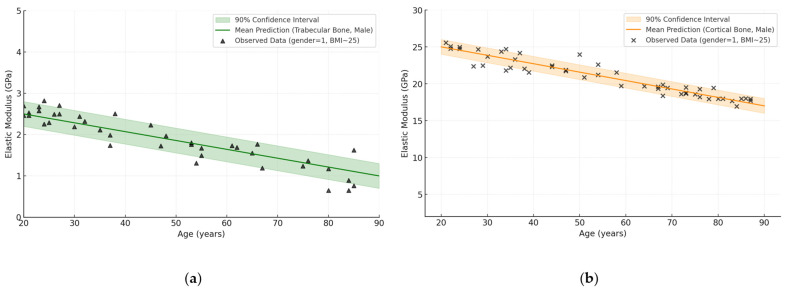
BNN prediction: dependence of elastic modulus (GPa) on age (years) for trabecular and cortical bone (male, BMI ≈ 25), based on reviewed dataset [38,39,43,46,49,53,55,56,57]: (**a**) trabecular bone; (**b**) cortical bone.

Continuing the theme of probabilistic modelling, BNN was also applied to a sample where sex was assumed to be 1 (male), with the same fixed BMI (BMI ≈ 25). As in the previous case, the raw data were summary measurements from papers [38,39,43,46,49,53,55,56,57]. The neural network was trained according to a similar scheme using a priori distributions and MCMC sampling, which allowed us to obtain an average prediction of elastic modulus with a confidence interval for the two categories of bone tissue (Figure 8).

Figure 8a shows that the average predicted trabecular elastic modulus in males is about 2.4 GPa at age 20 years and gradually decreases to about 1.0 GPa by age 80 years. For cortical bone (Figure 8b), values range from ~26 GPa at young age to ~18 GPa in the elderly. The shaded area reflects the 90% confidence interval of the model, showing the range of uncertainty. The observed points (markers) lie within the interval in most cases, indicating that the approximation is correct. Thus, the BNN demonstrates adequate ability to model age-related degradation of bone tissue elasticity taking into account biological variability.

**Figure 9:**
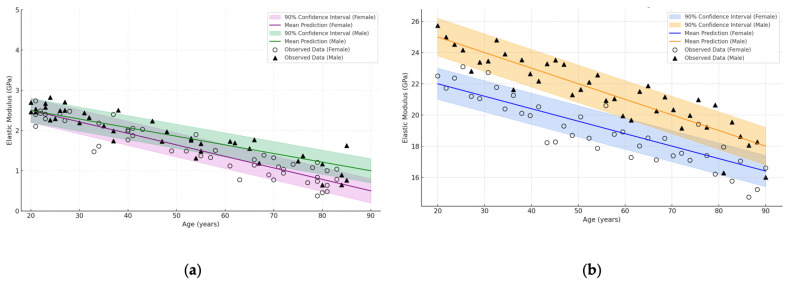
BNN prediction: bone elastic modulus (GPa) versus age (years) for females (○) and males (▲) (BMI ≈ 25) based on integrated literature data [38,39,43,46,49,53,55,56,57]: (**a**) trabecular bone; (**b**) cortical bone.

To clearly analyse the effect of gender on the age-related dynamics of bone tissue elastic properties, combined plots of BNN predictions for trabecular and cortical bone were constructed (Figure 9). In these models, gender was considered as an important differentiating factor, so two groups, female (○) and male (▲), were considered, with separate mean prediction lines and independent confidence intervals. The calculations were performed at a fixed BMI value (BMI ≈ 25) to minimise the effect of body weight on elastic characteristics.

The use of BNN not only allows us to predict the decrease in elastic modulus with age, but also to visualise the degree of uncertainty for each sex. The shaded areas show 90% confidence intervals, demonstrating where the model is “confident” in the prediction and where there is high variation in the biomechanical data. This approach allows multiple factors to be considered simultaneously and provides a powerful tool for predictive biomechanics and age risk assessment.

As can be seen from the graphs in Figure 9, with the same BMI value in both women and men, there is a clear tendency for bone elasticity modulus to decrease with age [38,39,43,46,49]. The values in the male sample remain higher throughout the age range, reflecting possible differences in bone mineral density and structure between the sexes [53,55]. This difference is particularly pronounced in cortical tissue, where the modulus of elasticity is on average 2–3 GPa higher in males. In the case of trabecular tissue, the differences are smoothed out, but the range of values also indicates a greater strength reserve in the male group [56,57]. BNN-based models can account for these inter-sex and age differences and visualise the level of uncertainty, which is critical for the tasks of personalised orthopaedics, fracture risk prediction and optimisation of implant design.

Overall, the data collected in the Results section indicate a significant decrease in Young’s modulus from young to old age for both cortical and cancellous tissue, although the severity of this trend may vary depending on the anatomical site, mineralisation level and marrow status [38,39,46,49].

At higher loading rates or confinement, a “hydraulic hardening” effect is evident [32,36,38], whereas at normal physiological rates of deformation, the contribution of the cerebral phase is limited.

The result of using Bayes’ theorem is the posterior probability of choosing the correct solution based on the observed data. Calculating the exact posterior distribution is quite difficult, sometimes impossible, especially with large data sets or complex models. In our study, the approximation of the function to provide a practical solution is reduced to the development of several equations for calculating the elastic modulus of bone tissues (denoted as E in Table 2) for the subsequent selection of the recommended elastic modulus of the finished implant, taking into account the biomechanical characteristics of bone tissue depending on age (young, old), sex (male, female) and anatomical zones of the human skeleton [38,39,46,49].

The obtained linear dependences emphasise a significant decrease in bone stiffness with age, especially pronounced in the cortical structures of the pelvis and femur. For trabecular tissue, the decrease is less pronounced due to its lower density and a different remodelling mode. The presented equations can be used to predict the mechanical properties of bone depending on the age of the patient when modelling osseointegration, designing implants and assessing fracture risk.

**Figure 10:**
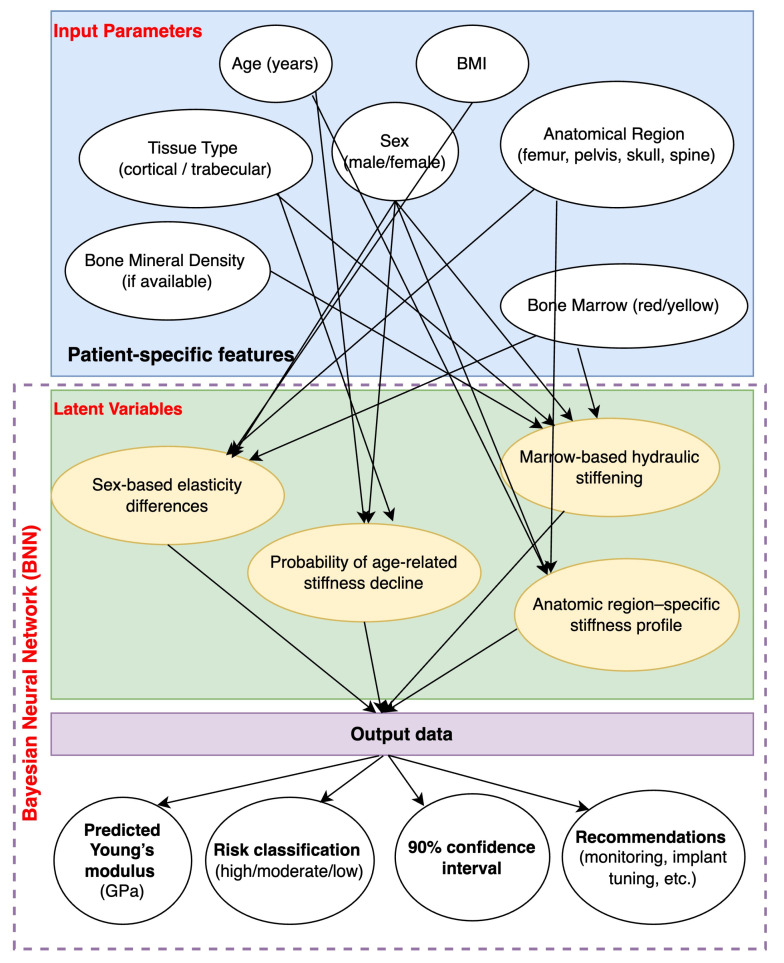
Structural and logical flowchart of the study of elastic properties of bone tissue: from biological factors and anatomical differences to modelling and prediction using BNN, based on cumulative findings from reviewed literature [6,12,25,27,28,29,30,32,33,37,39,43,46,49,53,55,56].

In order to comprehensively visualise the key stages of the study, factors affecting bone elastic properties, and the relationship between structural, anatomical, and age-related parameters, a summary flowchart was drawn up (Figure 10). It reflects the logic of the analysis, from baseline data and biological features to the application of probabilistic BNN modelling and interpretation of prognostic conclusions, based on cumulative findings from reviewed literature [6,12,25,27,28,29,30,32,33,37,39,43,46,49,53,55,56].

The proposed scheme (Figure 10) comprehensively illustrates the multifactorial nature of bone tissue elasticity, highlighting the key parameters—anatomical localisation, age, bone tissue type (cortical vs. trabecular), and bone marrow composition (red vs. yellow)—that collectively determine the mechanical behaviour of bone structures [6,12,27,30,32,33,39]. Each of these factors is known to influence stiffness and strength: for instance, trabecular bone is more prone to age-related degradation and loss of mechanical integrity compared to cortical bone [25,28,29,37].

By integrating BNN modelling into the workflow, we were able to quantitatively capture these biological dependencies and, crucially, assess the uncertainty associated with each prediction. The probabilistic nature of BNN allows not only the computation of average elastic modulus values but also the determination of confidence intervals, thereby offering a transparent measure of model reliability [43,46,49,53,55,56]. The width of these intervals serves as an indicator of underlying biological variability, experimental limitations, and the influence of factors such as sex differences and anatomical location.

Moreover, the modular nature of the developed scheme enables future expansion: additional layers of data—such as microstructural characteristics (e.g., collagen cross-linking, mineral crystal size) and molecular parameters (e.g., signalling pathways affecting bone remodelling)—can be incorporated to refine predictive performance. Advanced imaging modalities like μCT and nanoindentation, as well as molecular assays [13,16,34], will further enrich the input features and make the models more physiologically relevant.

In conclusion, this integrated probabilistic approach represents a significant advancement over traditional deterministic models. By embracing statistical uncertainty and biological heterogeneity, it becomes possible to create more robust and individualized predictions. This strategy has profound implications for clinical practice—particularly in the areas of prosthesis design, surgical planning, and fracture risk assessment—enabling the development of patient-specific implants and preventive interventions tailored to age, anatomical zone, and biomechanical needs [19,41,54].

Moreover, age-related bone loss and osteoporosis are critical aspects that should be taken into account when predicting mechanical properties. Numerous studies have confirmed that decreasing bone mineral density with age directly correlates with reduced stiffness and strength of both cortical and trabecular bone, significantly increasing fracture risk, especially in osteoporotic patients [13,25,28,29,37,53]. The integration of bone density parameters and osteoporosis status into future models will enable more accurate risk stratification and help to optimise implant design, surgical planning, and rehabilitation strategies.

## 4. Future Directions

Despite significant progress, the future of bone mechanics research requires the development of multiscale approaches that would link ex vivo data (i.e., obtained on bone samples extracted from the body and tested under laboratory conditions) to real physiological conditions [52]. Such methods could describe how changes at the level of collagen and mineral phases (especially with ageing) affect the overall strength of bone structure [53]. Additional attention should be paid to the pre-clinical evaluation of new materials for highly loaded skeletal zones, where the interaction between artificial implantation and native bone tissue is most critical [54].

The integration of biomechanical and biochemical variables remains an important direction, for example, in the modelling of bone-joint systems, where taking into account the state of cartilage and load distribution can significantly change the conclusions about bone strength and elasticity [55]. In such complex models, three-dimensional data (CT, MRI, X-ray) and precise measurements of the elastic properties of bone in different loading regimes play a special role [56]. The expansion of mathematical tools, including the use of approaches such as the extended finite element method for fracture analysis, will help to better understand the mechanisms of bone damage [60], including femoral neck fractures or endoprosthesis failure [57].

Finally, there is a growing need for hybrid computational frameworks that combine mechanistic (physics-based) models with probabilistic machine learning algorithms. This “two-pronged” approach will allow not only to identify statistical associations (e.g., between age, sex, BMI, and Young’s modulus) but also to test hypotheses about patterns of bone remodelling in osteopenia and osteoporosis. The implementation of Bayesian neural networks, which are able to quantify uncertainty, may be particularly useful when the number of observations is limited. Such multivariate analyses will provide a more complete understanding of bone remodelling and will also help to determine optimal strategies for rehabilitation and fracture prevention.

## 5. Conclusions

This review was performed similarly to the PRISMA methodology. To identify relevant articles, we consulted Scopus, eLibrary, PubMed, and Web of Science databases using key words in English: “bone elasticity”, “trabecular bone elasticity”, “cortical bone stiffness”, “bone marrow mechanics”, “Young’s modulus of bone”, “bone stiffness”, etc.

The comparison and selection of the collected publications showed that bone tissue elasticity is far from being a constant value: it is formed by a complex combination of anatomical localisation (hip, spine, skull, pelvis), age-related changes (young or old body), microstructure (cortical vs. trabecular) and bone marrow status. Young’s modulus ranges from 0.3 to 2.5 GPa for trabecular tissue and up to 30 GPa for cortical bone in young adults.

The trabecular component is the most vulnerable to age-related degradation: its elastic modulus can decrease by 40–60% after 60 years of age, which is associated with the loss of volume and integrity of the trabecular architecture. At the same time, the cortical layer shows a less dramatic decrease (usually within 10–30%), but remains key to the overall load-bearing capacity of the skeleton.

Particular attention is paid to gender differences: according to a number of studies and BNN models, women—especially in the postmenopausal period—are subject to a more dramatic decrease in the elastic characteristics of bone tissue, especially trabecular bone. In men, elastic modulus values are on average higher, especially at a young age, but also decrease with age. These differences emphasise the need for a gender-specific approach in clinical biomechanics. The composition of the bone marrow also plays an important role: if the semi-liquid phase is preserved, a “hydraulic strengthening” effect is possible, whereas defragmentation or washout of the marrow changes the test behaviour of the specimen.

To further illustrate the high variability, we used BNN, which not only generates mean predictions of elastic modulus based on age, sex and BMI, but also provides confidence intervals. This probabilistic approach allowed us to clearly demonstrate that even with the selected variables taken into account, there remains a marked level of uncertainty, reflected by the width of the “corridor” of predictions. This emphasises the importance of considering multiple factors and statistical fluctuations when modelling bone mechanics.

Thus, the results of the review and the BNN demonstration show that in order to develop reliable finite element models, improve orthopaedic implant design and accurately assess fracture risk, an integrated consideration of age, gender, anatomy, bone and bone marrow type and the use of probabilistic methods to quantify uncertainty is required. Such an integrated approach will allow for more accurate biomechanical predictions and ultimately better-informed clinical decisions in trauma and orthopaedics.

## Figures and Tables

**Table 1:** Summarised data on the elastic characteristics of bones according to different criteria.

Bone Region	Tissue Type	Age Group	Young’s Modulus (E_young’s_), GPa	Notes
Femur (shaft/diaphysis)	Cortical	The young ones (20–40 years old)	18–30	The most rigid layer; resistance to bending and stretching [6,7,17].
Femur (shaft/diaphysis)	Cortical	The elderly (65–70+ years).	10–25	Decrease in min. density, appearance of micropores; often associated with osteoporosis [14,15,16,17,18,19,20,21,22,23].
Femur (epiphysis/head/neck)	Trabecular.	The young ones (20–35 years old)	1–2	High porosity; actively involved in load cushioning [24,34,45,53].
Femur (epiphysis/head/neck)	Trabecular	The elderly (60–70+ years).	0.5–1.2	Osteoporotic changes, reduction in the number and thickness of trabeculae are possible.
Pelvis (wing of the ilium)	Cortical	The young ones (20–40 years old)	15–20	Somewhat lower density than femoral, but still quite stiff [42,43,44,45,46].
Pelvis (wing of the ilium)	Cortical	The elderly (60–70+ years).	10–15	Decrease in min. density in the cortical layer, age-related porosity [21,22,23,24,25,26,27,28]
Pelvis (wing of the ilium)	Trabecular	The young ones (20–40 years old)	1.5–2	Fairly high elasticity; important for pelvic energy capacity [38,39,40,41,42,43,44,45]
Pelvis (wing of the ilium)	Trabecular	The elderly (60–70+ years).	0.8–1.2	Diminished performance, osteopenia/osteoporosis reduce load-bearing properties [23].
Vertebrae (vertebral bodies)	Trabecular	Young/middle-aged	1–2	Axial load support; reduced ~2-fold in osteopenia [19,20,21,22,23,24,25,26,27,28,29,30,31,32].
Vertebrae (vertebral bodies)	Trabecular	The elderly (70+ years).	0.3–0.5	A serious loss of strength (−30–50%) is possible by old age [20,21,22,23,24,25,26,27,28,29].
Cranium (skull, cortical layer).	Cortical (outer/inner plates)	Adults (30–60 years old).	7–15	Thin layer, different pattern of collagen fibres, values may be lower [9,10,11,12,13,14,15].
Cranium.	Trabecular (spongy layer)	Different age categories	0.2–1.5	Large variations in density are observed with age-related changes [21,22].

**Table 2:** Modulus of elasticity of bone tissues as a function of age 20–75 years.

№.	Types of Bone Tissue	Modulus of Elasticity (GPa)
1	Cortical (hips, shoulders, tibia)	E = 29.571 − 0.1857x
2	Cortical (skull)	E = 13.829 − 0.0943x
3	Cortical (pelvis)	E = 29.5 − 0.4x
4	Trabecular (hips, vertebrae)	E = 1.979 − 0.0243x
5	Trabecular (pelvis)	E = 2.95 − 0.04x
6	Trabecular (total range)	E = 8.0143 − 0.1171x
Note: x-age from 20 to 75 years old.		

## Data Availability

The data presented in this study are available from the corresponding author upon reasonable request.

## Abbreviations

The following abbreviations are used in this manuscript:

BMI	Body Mass Index
BNN	Bayesian Neural Network
CT	Computed Tomography
E	Modulus of Elasticity
FEA	Finite Element Analysis
GPa	Gigapascal
PINNs	Physics-Informed Neural Networks
SDE	Stochastic Differential Equations

## Appendix A

**Table A1:** Assessment of the completeness of the coverage of bone elastic characteristics (★ indicates presence of relevant content in the section; 0 indicates absence).

Author (Year)	Abstract	Introduction	Methodology	Results	Discussion	Others	Score
1. Rosa et al. (2022) [1]	★	★	★	★	★	0	5/6
2. Niu et al. (2023) [2]	★	★	★	★	0	★	5/6
3. Hart et al. (n.d.) [3]	★	★	0	★	0	★	4/6
4. Sansalone et al. (2012) [15]	★	★	★	★	★	0	5/6
5. Pritchard et al. (2013) [16]	★	0	★	★	★	0	4/6
6. Pope et al. (1974) [61]	★	★	0	★	0	★	4/6
7. Cole & Van Der Meulen (2020) [5]	★	★	★	0	★	★	5/6
8. Martin (1991) [17]	★	0	0	★	★	★	4/6
9. Goldstein (1987) [18]	★	★	★	★	★	0	5/6
10. Augat et al. (1998) [19]	★	★	0	★	0	★	4/6
11. Bouxsein (2001) [20]	★	★	★	★	★	★	6/6
12. Gomez & Nahum (2002) [6]	★	0	★	★	★	★	5/6
13. Morgan et al. (2018) [7]	★	★	0	★	★	0	4/6
14. Ingole et al. (2021) [21]	★	★	★	★	0	★	5/6
15. Battafarano et al. (2021) [22]	0	★	★	★	★	★	5/6
16. Garcia-Giner et al. (2021) [23]	★	★	0	★	0	0	3/6
17. Álvarez-Lloret et al. (2021) [24]	★	★	★	★	★	0	5/6
18. Nefjodovs et al. (2023) [25]	0	★	0	★	★	★	4/6
19. Gasik et al. (2021) [26]	★	★	★	★	0	0	4/6
20. Grill et al. (2025) [27]	★	★	0	★	★	0	4/6
21. Bhattarai et al. (2018) [28]	0	★	★	★	0	★	4/6
22. Ferraz (2024) [29]	★	★	0	★	★	★	5/6
23. Castro-Franco et al. (2020) [30]	★	0	★	★	★	★	5/6
24. Łuczak et al. (2024) [31]	★	0	0	★	★	★	4/6
25. Costăchescu et al. (2023) [32]	★	★	★	★	★	0	5/6
26. Zhao et al. (2023) [4]	0	★	0	★	0	★	3/6
27. Zhao et al. (2018) [8]	★	★	★	★	0	★	5/6
28. Turner et al. (1999) [9]	★	0	0	★	★	0	3/6
29. Ashman et al. (1987) [33]	★	★	★	0	0	★	4/6
30. Sansalone et al. (2010) [15]	★	★	0	★	★	★	5/6
31. Hamandi et al. (2021) [35]	★	★	★	★	0	★	5/6
32. Nikolov & Raabe (2008) [36]	★	0	0	★	★	0	3/6
33. Salguero et al. (2014) [37]	★	★	★	0	★	0	4/6
34. Blázquez-Carmona et al. (2024) [10]	★	0	★	★	0	★	4/6
35. Katz et al. (1984) [38]	★	★	0	★	★	★	5/6
36. Turek et al. (2025) [39]	★	★	★	★	0	0	4/6
37. Stojković et al. (2023) [11]	★	0	0	★	★	★	4/6
38. Ibrahim et al. (2020) [40]	★	★	★	0	★	0	4/6
39. Ibrahim et al. (2024) [12]	0	★	★	★	★	0	4/6
40. Sanz et al. (2023) [13]	★	0	0	★	★	★	4/6
41. Bazyar et al. (2023) [41]	★	★	★	0	★	★	5/6
42. Baleani et al. (2024) [42]	★	★	0	★	0	★	4/6
43. Lin & Kang (2021) [43]	★	★	★	★	★	★	6/6
44. Hamandi & Goswami (2021) [35]	★	0	★	0	★	★	4/6
45. Hamandi & Goswami (2022) [46]	★	★	0	★	★	0	4/6

**Table A2:** Brief study characteristics, sample demographics, key findings or summary.

Experimental Methods	Authors, Year of Publication	Journal of Publication	Types of Specimens	Numbers of Specimens	Gender	Age	Anatomic Sites	Main Findings or Summaries
Mechanical tests (3-point bending, compression)	1. Rosa et al. (2022) [1]	Applied Sciences	Cylindrical/rectangular sections from long bones (in vitro)	30	Both	30–65	Femoral diaphysis, tibial cortex.	Demonstrates that bone is an outstanding natural “composite”, highlighting the synergy between collagen matrix and mineral phase; mechanical performance correlates with porosity and fibre orientation.
Ultrasound + CT correlation	2. Niu et al. (2023) [2]	Journal of Functional Biomaterials	Intact cadaveric bones (ex vivo)	25	Mixed	40–80	Vertebral bodies	Emphasises the role of microstructural features in modulating bone’s elastic modulus; proposes new analysis approaches combining imaging and acoustic velocity measures.
Scanning electron microscopy (SEM) + hardness testing	3. Hart et al. (n.d.) [3]	- (Online article/PMC)	Human cortical bone slices	12	Not specified	20–45	Femoral midshaft	Biological basis of bone strength (anatomy and physiology) analysing microcracks on SEM images; highlights measuring different mechanical parameters (microhardness, porosity).
Nanoindentation + morphological assessment	4. Sansalone et al. (2012) [15]	Bone	Femoral neck trabecular bone (human)	18	Both	40–75	Human femoral neck	Demonstrates that distribution of mineralisation significantly affects local mechanical properties in the femoral neck; higher heterogeneity→ different local stiffness values.
DXA-based + ex vivo destructive testing	5. Pritchard et al. (2013) [16]	Bone	Bones from diabetic vs. non-diabetic patients	20	Not specified	50–70	Knee osteoarthritis and T2D bone surfaces	Observed elevated and more homogeneous mineralisation in type 2 diabetes with osteoarthritis, indicating potential differences in stiffness compared to osteoarthritis alone.
Literature review + mechanical perspective	6. Pope et al. (1974) [61]	CRC Press eBooks	-	-	-	-	General overview	Summarises fundamental mechanical properties of bone, focusing on how composition and hierarchical structure govern the elastic/plastic behaviour.
Finite Element Analysis (FEA) + micro-CT	7. Cole & Van Der Meulen (2020) [5]	Contemporary Endocrinology	Micro-CT scanned mouse tibiae, in silico simulation	16	Both	Various (adult mice)	Tibial cortical/trabecular	Demonstrates how FEA can link changes in bone geometry to predicted strain distribution, relevant for endocrine disorders affecting bone.
Statistical analysis of mechanical tests	8. Martin (1991) [17]	Journal of Biomechanics	Various literature data reanalysis	-	-	-	Multiple (long bones)	Identifies determinants of mechanical properties (mineral density, collagen cross-links) through reanalysis of prior datasets; highlights role of microdamage in older bones.
In vitro tension/compression	9. Goldstein (1987) [18]	Journal of Biomechanics	Bovine/human trabecular bone samples	28	-	-	Distal femur, vertebrae	The mechanical properties of trabecular bone heavily depend on anatomic location and function; cortical-trabecular transitions also matter.
Ultrasonic measurement of anisotropy	10. Augat et al. (1998) [19]	Medical Engineering & Physics	Human/mammalian trabecular bone blocks	20	Both	30–75	Distal radius, proximal tibia.	Demonstrates anisotropy in the elastic modulus of trabecular bone from different regions, correlating with load direction.
Review: age-related fractures	11. Bouxsein (2001) [20]	Elsevier eBooks	-	-	-	-	General (osteoporotic fractures)	Discusses the link between mechanical properties and fracture risk in older adults, emphasizing that material properties + architecture synergy is key to understanding fragility.
Comparative approach: bone vs. trauma	12. Gomez & Nahum (2002) [6]	Springer eBooks	-	-	-	-	Various (long bones, spongy bone)	Biomechanics of bone in accidental injury context; highlights energy absorption capacity in relation to microstructure and orientation.
Multiscale mechanical analysis	13. Morgan et al. (2018) [7]	Annual Review of Biomedical Engineering	Human trabecular + cortical data compilation	Large meta-study	Both	20–80 (various)	Multiple (femur, tibia, spine)	Summarises healthy/diseased states; indicates that while architecture drastically changes with osteoporosis, lamellar-level properties remain comparatively stable.
Sintered hydroxyapatite mechanical test	14. Ingole et al. (2021) [21]	Minerals	Hydroxyapatite-based materials (compression, HPC)	-	-	-	Synthetic bone regeneration setups	Found that nanostructured hydroxyapatite can match certain mechanical benchmarks of bone, focusing on bone tissue engineering applications.
Bone graft/tissue engineering review	15. Battafarano et al. (2021) [22]	International Journal of Molecular Sciences	-	-	-	-	Highly loaded skeletal sites	Summarises strategies for bone regeneration with biomaterials; mechanical stability is essential in sites subject to high loads.
Nanoscale imaging	16. Garcia-Giner et al. (2021) [23]	Applied Sciences	Human bone pathologies (micro-CT + SEM)	10	Both	50–70	Various (skull, femur)	Reviews imaging and analysis methods for bone pathologies at the nanoscale, discussing correlation with reduced mechanical competence.
Metal contamination and mineral properties	17. Álvarez-Lloret et al. (2021) [24]	Minerals	Alveolar bone under lead exposure	8	Both	30–45 (animal model)	Alveolar bone	Shows that chronic lead exposure alters mineral properties and mechanical stability, which may serve as a model for human alveolar bone changes.
Wood as bone surrogate	18. Nefjodovs et al. (2023) [25]	Journal of Functional Biomaterials	Wood-based surrogates in mechanical tests	-	-	-	-	Reviews feasibility of using wood as a renewable material to mimic certain structural aspects of bone (porosity, anisotropy); discusses limitations for direct mechanical comparison.
Implant design approaches	19. Gasik et al. (2021) [26]	Materials	Dental implants (in vivo + in silico)	-	-	-	Mandibular bone	Analyses how bone-implant interface mechanics vary by bone density and location; suggests consideration of both cortical and trabecular compartments for robust design.
Bone regeneration (mini-review)	20. Grill et al. (2025) [27]	Bioengineering	-	-	-	-	Various (general approach)	Short overview on bone regeneration techniques; mechanical perspectives on scaffolds and host bone integration.
Electrospun fibrous materials	21. Bhattarai et al. (2018) [28]	Membranes	Fibrous polymer scaffolds (experimental)	-	-	-	Tissue engineering applications	Discusses mechanical properties of natural vs. synthetic electrospun scaffolds for bone repair.
Bone substitute materials	22. Ferraz (2024) [29]	International Journal of Molecular Sciences	Biomaterials in bone TE (ex vivo/in vivo)	-	-	-	Various load-bearing sites	Emphasises composite scaffolds that replicate bone’s mechanical environment, focusing on mechanical + biological synergy.
Proximal humerus characterisation	23. Castro-Franco et al. (2020) [30]	Applied Sciences	Humerus mechanical property evaluation	12	Both	45–70	Proximal humerus	Reviews the proximal humerus in biomechanical studies, highlights incomplete data on anisotropy and a need for standardised protocols.
Emerging technologies in bone repair	24. Łuczak et al. (2024) [31]	International Journal of Molecular Sciences	-	-	-	-	Future bone regeneration devices	Summary of upcoming biomaterials, 3D printing, and mechanical testing strategies for advanced bone scaffolds.
Screw osteointegration	25. Costăchescu et al. (2023) [32]	Materials	Screw pull-out mechanical tests	40	Both	40–65	Femoral condyles + tibial plateau	Concludes that enhancing biomechanical resistance to pull-out effect depends on correct match of screw geometry and local bone density.
Bone organoids (review)	26. Zhao et al. (2023) [4]	Advanced Healthcare Materials	In vitro bone organoid models	-	-	-	-	Highlights recent advances and future challenges in the development of bone organoids; discusses mechanical environment’s importance in organoid maturity.
Compression test standardisation	27. Zhao et al. (2018) [8]	Bone and Joint Research	Human cortical bone blocks	40	Both	55–80 years	Femoral diaphysis (cortical regions)	The article emphasises the significant variability in bone stiffness results due to differences in testing protocols (specimen geometry, end-face quality, specimen orientation). The authors propose a standardised approach to compression testing to reduce variability and increase comparability between different studies.
Elastic properties (microscopic)	28. Turner et al. (1999) [9]	Journal of Biomechanics	Human cortical + trabecular (micro-tests)	-	Both	20–70	Various (femur, tibia)	Finds that, at the microscopic level, cortical and trabecular tissues can show surprisingly similar elastic properties, if tested under consistent protocols.
Ultrasonic technique	29. Ashman et al. (1987) [33]	Journal of Biomechanics	Cancellous bone, ultrasonic velocity	20	-	-	Distal femur, proximal tibia.	Concludes that acoustic-based methods can measure elastic properties of cancellous bone, showing site-specific differences.
Heterogeneous anisotropic elastic properties	30. Sansalone et al. (2010) [15]	Journal of Biomechanics	Human femoral bone cubes (10 mm)	14	Both	50–80	Femoral diaphysis/neck	Found significant anisotropy at multiple scales; link to remodelling pattern and local mineralisation.
Retrospective evaluation (anisotropic)	31. Hamandi et al. (2021) [35]	Bioengineering	Literature data and finite element comparisons	-	-	-	Various (long bones)	Proposed a framework for matching anisotropic material behaviour vs. elastic, elastic-plastic, and hyper-elastic properties in bone.
Hierarchical modelling	32. Nikolov & Raabe (2008) [36]	Biophysical Journal	Multiscale model (submicron)	-	-	-	Collagen-mineral arrangement	Shows that extrafibrillar mineralisation drastically affects submicron elasticity; hierarchical modelling bridging nano to macro scale.
Micromechanical modelling	33. Salguero et al. (2014) [37]	Journal of Biomechanics	Cortical bone (modelling)	-	-	-	Mid-diaphyseal cortical (human)	Accounted for anisotropy of dense tissue in micromechanical modelling, concluding that orientation of vascular channels is a key factor.
Woven bone mechanical properties	34. Blázquez-Carmona et al. (2024) [10]	Frontiers in Bioengineering and Biotechnology	Healing woven bone specimens (animal)	20	Both	~2–3 months post-surgery	Femoral defect model	Observes how structural and chemical properties of woven bone under healing correlate with local variations in elastic modulus.
Remodelling effect	35. Katz et al. (1984) [38]	Calcified Tissue International	Various bone sites (thin sections)	15	Mixed	~40–65	Femur, tibia.	Argues that remodelling significantly alters elastic properties; local histological changes lead to different stiffness regions.
Aspect-Related mechanical testing	36. Turek et al. (2025) [39]	Applied Sciences	Metacarpal bone of mares (equine)	28	Female (mares)	Adult equines	3rd metacarpal bone	Shows that cortical bone mechanical properties in horses vary by aspect and that consistent anatomic referencing is crucial.
Electron Beam Melting (EBM) lattice	37. Stojković et al. (2023) [11]	Metals	3D-printed Ti6Al4V scaffolds	16	-	-	Anatomically shaped lattice bone scaffold	Noted adjustable elasticity by controlling lattice geometry; relevant for load-bearing implants.
Hardness and collagen	38. Ibrahim et al. (2020) [40]	Journal of Functional Biomaterials	Bone microhardness test (Vickers), ex vivo	10	Not specified	~30–60	Femoral cortical/trabecular	Identifies hardness as an important indicator of bone quality; collagen plays a major role in local hardness variations.
Viscoelastic deformation	39. Ibrahim et al. (2024) [12]	Journal of Functional Biomaterials	Vickers hardness + time-dependent analysis	8	Both	40–65	Femoral diaphysis	Discovers a novel mechanism of time-dependent (viscoelastic) deformation under indentation, reinforcing the significance of collagen matrix.
Juvenile bone properties	40. Sanz et al. (2023) [13]	Materials	Juvenile long bones (mechanical tests)	25	Both (juvenile)	5–15 years	Tibia, femur	Variation in mechanical behaviour found as a function of age in juvenile bones; potential collision/trauma analysis.
Finite Element modelling (femur)	41. Bazyar et al. (2023) [41]	Biomechanics	Human femur FE modelling + material property assignment	-	Both	55–70 (cadaveric)	Proximal femur	Overview of property definition in FE models of the femur; emphasises how small changes in anisotropy input can alter simulation outcomes.
Tensile Yield Strain	42. Baleani et al. (2024) [42]	Bioengineering	Femoral diaphysis (human) testing	14	Mixed	35–70	Human femoral diaphysis	Suggests that tensile yield strain remains constant among healthy adults, providing a reference baseline for future mechanical evaluations.
Uniaxial tensile test review	43. Lin & Kang (2021) [43]	Materials	Literature review + some new tests	~40	Mixed	20–60	Long bones, cortical shell	Provides practical guidelines on measuring stress–strain curves of compact bone under uniaxial tension; notes pitfalls and recommended protocols.
Anisotropic vs. Hyperelastic	44. Hamandi & Goswami (2021) [35]	Bioengineering	Literature-based + new computational approach	-	-	-	Various (long bones)	Developed a framework to compare anisotropic, elastic-plastic, and hyperelastic models for bone, concluding anisotropy is essential for accuracy.
Hierarchical structure (nano-level)	45. Hamandi & Goswami (2022) [46]	Bioengineering	Bone hierarchical data (nanoindentation, micro-CT)	10	Mixed	30–70	Femoral cortical, tibial cortical.	Emphasises that hierarchical structure and nano-level features significantly influence macro-level mechanical response.

## References

[1] Rosa N., Moura M.F.S.F., Olhero S., Simoes R., Magalhães F.D., Marques A.T., Ferreira J.P.S., Reis A.R., Carvalho M., Parente M. (2022). Bone: An outstanding composite material. Appl. Sci..

[2] Niu Y., Du T., Liu Y. (2023). Biomechanical characteristics and analysis approaches of bone and bone substitute materials. J. Funct. Biomater..

[3] Hart N.H., Newton R.U., Tan J., Rantalainen T., Chivers P., Siafarikas A., Nimphius S. (2020). Biological basis of bone strength: Anatomy, physiology and measurement. J. Musculoskelet. Neuronal Interact..

[4] Zhao D., Saiding Q., Li Y., Tang Y., Cui W. (2023). Bone organoids: Recent advances and future challenges. Adv. Healthc. Mater..

[5] Cole J.H., Van Der Meulen M.C.H. (2020). Biomechanics of bone. Contemporary Endocrinology.

[6] Gomez M.A., Nahum A.M. (2002). Biomechanics of bone. Accidental Injury: Biomechanics and Prevention.

[7] Morgan E.F., Unnikrisnan G.U., Hussein A.I. (2018). Bone mechanical properties in healthy and diseased states. Annu. Rev. Biomed. Eng..

[8] Zhao S., Arnold M., Ma S., Abel R.L., Cobb J.P., Hansen U., Boughton O. (2018). Standardizing compression testing for measuring the stiffness of human bone. Bone Jt. Res..

[9] Turner C.H., Rho J., Takano Y., Tsui T.Y., Pharr G.M. (1999). The elastic properties of trabecular and cortical bone tissues are similar: Results from two microscopic measurement techniques. J. Biomech..

[10] Blázquez-Carmona P., Mora-Macías J., Pajares A., Mármol Á., Reina-Romo E. (2024). On the influence of structural and chemical properties on the elastic modulus of woven bone under healing. Front. Bioeng. Biotechnol..

[11] Stojković J.R., Stojković M., Turudija R., Aranđelović J., Marinkovic D. (2023). Adjustable elasticity of anatomically shaped lattice bone scaffold built by electron beam melting TI6AL4V powder. Metals.

[12] Ibrahim A., Jiang Z., Shirvani K., Dalili A., Hamid Z.A. (2024). A Novel Viscoelastic Deformation Mechanism Uncovered during Vickers Hardness Study of Bone. J. Funct. Biomater..

[13] Sanz C.V., Rodríguez I.V., Forriol F., Tejado E., Lopez-Valdes F.J. (2023). Variation in juvenile long bone properties as a function of age: Mechanical and compositional characterisation. Materials.

[14] García-Vilana S., Sánchez-Molina D., Abdi H. (2025). Acoustic Emission in Bone Biomechanics: A Comprehensive review of mechanical properties and predictive damage modelling. Sensors.

[15] Sansalone V., Bousson V., Naili S., Bergot C., Peyrin F., Laredo J.D., Haïat G. (2012). Anatomical distribution of the degree of mineralisation of bone tissue in human femoral neck: Impact on biomechanical properties. Bone.

[16] Pritchard J.M., Papaioannou A., Tomowich C., Giangregorio L.M., Atkinson S.A., Beattie K.A., Adachi J.D., DeBeer J., Winemaker M., Avram V. (2013). Bone mineralisation is elevated and less heterogeneous in adults with type 2 diabetes and osteoarthritis compared to controls with osteoarthritis alone. Bone.

[17] Martin R.B. (1991). Determinants of the mechanical properties of bones. J. Biomech..

[18] Goldstein S.A. (1987). The mechanical properties of trabecular bone: Dependence on anatomic location and function. J. Biomech..

[19] Augat P., Link T., Lang T.F., Lin J.C., Majumdar S., Genant H.K. (1998). Anisotropy of the elastic modulus of trabecular bone specimens from different anatomical locations. Med. Eng. Phys..

[20] Bouxsein M.L. (2001). Biomechanics of Age-Related Fractures. Osteoporosis.

[21] Ingole V.H., Ghule S.S., Vuherer T., Kokol V., Ghule A.V. (2021). Mechanical properties of differently nanostructured and High-Pressure compressed Hydroxyapatite-Based materials for bone tissue regeneration. Minerals.

[22] Battafarano G., Rossi M., De Martino V., Marampon F., Borro L., Secinaro A., Del Fattore A. (2021). Strategies for bone regeneration: From graft to tissue engineering. Int. J. Mol. Sci..

[23] Garcia-Giner V., Han Z., Giuliani F., Porter A.E. (2021). Nanoscale imaging and analysis of bone pathologies. Appl. Sci..

[24] Álvarez-Lloret P., Benavides-Reyes C., Lee C.M., Martínez M.P., Conti M.I., Rodríguez-Navarro A.B., González-López S., Perez-Huerta A., Terrizzi A.R. (2021). Chronic lead exposure alters mineral properties in alveolar bone. Minerals.

[25] Nefjodovs V., Andze L., Andzs M., Filipova I., Tupciauskas R., Vecbiskena L., Kapickis M. (2023). Wood as a possible renewable material for bone implants—Literature review. J. Funct. Biomater..

[26] Gasik M., Lambert F., Bacevic M. (2021). Biomechanical properties of bone and mucosa for design and application of dental implants. Materials.

[27] Grill S.L., Brouillet F., Drouet C. (2025). Bone Regeneration: Mini-Review and Appealing Perspectives. Bioengineering.

[28] Bhattarai D.P., Aguilar L.E., Park C.H., Kim C.S. (2018). A review on properties of natural and synthetic based electrospun fibrous materials for bone tissue engineering. Membranes.

[29] Ferraz M.P. (2024). An overview on the big players in bone tissue engineering: Biomaterials, scaffolds and cells. Int. J. Mol. Sci..

[30] Castro-Franco A.D., Mendoza-Muñoz I., González-Ángeles Á., Cruz-Sotelo S.E., Castañeda A.M., Siqueiros-Hernández M. (2020). Trends in the characterisation of the proximal humerus in Biomechanical Studies: A review. Appl. Sci..

[31] Łuczak J.W., Palusińska M., Matak D., Pietrzak D., Nakielski P., Lewicki S., Grodzik M., Szymański Ł. (2024). The Future of Bone Repair: Emerging technologies and biomaterials in bone regeneration. Int. J. Mol. Sci..

[32] Costăchescu B., Niculescu A.-G., Grumezescu A.M., Teleanu D.M. (2023). Screw Osteointegration—Increasing biomechanical resistance to Pull-Out effect. Materials.

[33] Ashman R.B., Corin J.D., Turner C.H. (1987). Elastic properties of cancellous bone: Measurement by an ultrasonic technique. J. Biomech..

[34] Sansalone V., Naili S., Bousson V., Bergot C., Peyrin F., Zarka J., Laredo J.D., Haïat G. (2010). Determination of the heterogeneous anisotropic elastic properties of human femoral bone: From nanoscopic to organ scale. J. Biomech..

[35] Hamandi F., Tsatalis J.T., Goswami T. (2021). Retrospective Evaluation and Framework Development of Bone Anisotropic Material Behaviour Compared with Elastic, Elastic-Plastic, and Hyper-Elastic Properties. Bioengineering.

[36] Nikolov S., Raabe D. (2008). Hierarchical modelling of the elastic properties of bone at submicron scales: The role of extrafibrillar mineralization. Biophys. J..

[37] Salguero L., Saadat F., Sevostianov I. (2014). Micromechanical modelling of elastic properties of cortical bone accounting for anisotropy of dense tissue. J. Biomech..

[38] Katz J.L., Yoon H.S., Lipson S., Maharidge R., Meunier A., Christel P. (1984). The effects of remodelling on the elastic properties of bone. Calcif. Tissue Int..

[39] Turek B., Mikułowski G., Szara T., Dołasiński M., Jasiński T., Domino M. (2025). Aspect-Related mechanical properties of the cortical bone in the third metacarpal bone of mares. Appl. Sci..

[40] Ibrahim A., Magliulo N., Groben J., Padilla A., Akbik F., Hamid Z.A. (2020). Hardness, an important indicator of bone quality, and the role of collagen in bone hardness. J. Funct. Biomater..

[41] Bazyar P., Baumgart A., Altenbach H., Usbeck A. (2023). An overview of selected material properties in finite element modelling of the human femur. Biomechanics.

[42] Baleani M., Erani P., Acciaioli A., Schileo E. (2024). Tensile Yield Strain of Human Cortical Bone from the Femoral Diaphysis Is Constant among Healthy Adults and across the Anatomical Quadrants. Bioengineering.

[43] Lin C.-Y., Kang J.-H. (2021). Mechanical properties of compact bone defined by the Stress-Strain curve measured using uniaxial tensile Test: A concise review and Practical guide. Materials.

[44] Solarino G., Vicenti G., Picca G., Rifino F., Carrozzo M., Moretti B. (2016). A review of gender differences in hip fracture anatomy, morbidity, mortality and function. Ital. J. Gend.-Specif. Med.

[45] Oefner C., Riemer E., Funke K., Werner M., Heyde C.-E., Schoenfelder S. (2021). Determination of Anisotropic Elastic Parameters from Morphological Parameters of Cancellous Bone for Osteoporotic Lumbar Spine. Med. Biol. Eng. Comput..

[46] Hamandi F., Goswami T. (2022). Hierarchical structure and properties of the bone at nano level. Bioengineering.

[47] Kontomaris S.V., Stylianou A., Chliveros G., Malamou A. (2023). Determining spatial variability of elastic properties for biological samples using AFM. Micromachines.

[48] Sun C., Dong E., Chen J., Zheng J., Kang J., Jin Z., Liu C., Wang L., Li D. (2022). The promotion of mechanical properties by bone ingrowth in Additive-Manufactured Titanium Scaffolds. J. Funct. Biomater..

[49] Zeng Y., Meng Q., Chen Y., Zou D., Tao L. (2022). Age-Related study and collision response of material properties of long bones in Chinese pedestrian lower limbs. Appl. Sci..

[50] Singh N., Trajkovski A., Trajkovski J., Kunc R., Matas J.F.R. (2025). A pilot study on the Age-Dependent, biomechanical properties of longitudinal ligaments in the human cervical spine. Bioengineering.

[51] Aurégan J.-C., Bosser C., Bachy-Razzouk M., Bensidhoum M., Hoc T. (2023). In vivo assessment of skin surface pattern: Exploring its potential as an indicator of bone biomechanical properties. Bioengineering.

[52] Kulić M., Bagavac P., Bekić M., Krstulović-Opara L. (2024). Ex vivo biomechanical bone testing of pig femur as an experimental model. Bioengineering.

[53] Leng H., Reyes M.J., Dong X.N., Wang X. (2013). Effect of age on mechanical properties of the collagen phase in different orientations of human cortical bone. Bone.

[54] De Lacerda Schickert S., Van Den Beucken J.J.J.P., Leeuwenburgh S.C.G., Jansen J.A. (2020). Pre-Clinical evaluation of biological bone substitute materials for application in highly loaded skeletal sites. G.; Jansen, J.A. Pre-Clinical evaluation of biological bone substitute materials for application in highly loaded skeletal sites. Biomolecules.

[55] Belluzzi E., Todros S., Pozzuoli A., Ruggieri P., Carniel E.L., Berardo A. (2023). Human Cartilage Biomechanics: Experimental and Theoretical Approaches towards the Identification of Mechanical Properties in Healthy and Osteoarthritic Conditions. Processes.

[56] Pallua J.D., Putzer D., Jäger E., Degenhart G., Arora R., Schmölz W. (2022). Characterizing the mechanical behaviour of bone and bone surrogates in compression using PQCT. Materials.

[57] Nguyen H.-Q., Nguyen T.-N.-T., Pham T.-Q.-D., Nguyen V.-D., Van Tran X., Dao T.-T. (2021). Crack Propagation in the Tibia Bone within Total Knee Replacement Using the eXtended Finite Element Method. Appl. Sci..

[58] Glatt V., Canalis E., Stadmeyer L., Bouxsein M.L. (2007). Age-Related Changes in Trabecular Architecture Differ in Female and Male C57BL/6J Mice. J. Bone Miner. Res..

[59] Ngartera L., Issaka M.A., Nadarajah S. (2024). Application of Bayesian Neural Networks in Healthcare: Three Case Studies. Mach. Learn. Knowl. Extr..

[60] Mukhtar A.M., Könke C. (2011). Fracture mechanics and micro crack detection in bone: A short communication. Conference Medical Device Materials V. Novelty.

[61] Pope M.H., Outwater J.O. (1974). Mechanical Properties of Bone as a Function of Position and Orientation. J. Biomech..

